# Comparing the Developmental Toxicity Delay and Neurotoxicity of Benzothiazole and Its Derivatives (BTHs) in Juvenile Zebrafish

**DOI:** 10.3390/toxics12050341

**Published:** 2024-05-07

**Authors:** Xiaogang Yin, Lei Wang, Lianshan Mao

**Affiliations:** 1 College of Chemical Engineering, Nanjing Forestry University, Nanjing 210037, China; yinxiaogang@njfu.edu.cn; 2 College of Agricultural Science and Engineering, Hohai University, Nanjing 210098, China; wl18260579972@gmail.com

**Keywords:** benzothiazole, zebrafish, neurotoxicity, behavioral science, oxidative stress

## Abstract

In this study, a semi-static water exposure method was employed to investigate the early developmental and neurotoxic effects of four benzothiazole substances (BTHs), namely benzothiazole (BTH), 2-mercaptobenzothiazole (MBT), 2-hydroxybenzothiazole (BTON), and 2-aminobenzothiazole (2-ABTH), on zebrafish at an equimolar concentration of 10 μM. The findings revealed that all four BTHs exerted certain impacts on early development in zebrafish. MBT stimulated spontaneous movement in juvenile zebrafish, whereas BTON inhibited such movements. Moreover, all four BTHs hindered the hatching process of zebrafish larvae, with MBT exhibiting the strongest inhibition at 24 h post-fertilization (hpf). Notably, MBT acted as a melanin inhibitor by suppressing melanin production in juvenile zebrafish eyes and weakening phototaxis. Additionally, both BTH and BTON exhibited significantly lower speeds than the control group and other test groups under conditions without bright field stimulation; however, their speeds increased to average levels after percussion stimulation, indicating no significant alteration in motor ability among experimental zebrafish groups. Short-term exposure to these four types of BTHs induced oxidative damage in zebrafish larvae; specifically, BTH-, MBT-, and BTON-exposed groups displayed abnormal expression patterns of genes related to oxidative damage. Exposure to both BTH and MBT led to reduced fluorescence intensity in transgenic zebrafish labeled with central nervous system markers, suggesting inhibition of central nervous system development. Furthermore, real-time quantitative PCR results demonstrated abnormal gene expression associated with neural development. However, no significant changes were observed in 2-ABTH gene expression at this concentration. Overall findings indicate that short-term exposure to BTHs stimulates neurodevelopmental gene expression accompanied by oxidative damage.

## 1. Introduction

Benzothiazole analogs (BTHs) are a group of aromatic heterocyclic compounds with various industrial and commercial applications, such as rubber manufacturing, dyes, pharmaceuticals, and pesticides. Consequently, BTHs are released directly or indirectly into the natural environment through various channels. Direct sources include industrial wastewater of dye factories, pharmaceutical factories, and pesticide factories, which is discharged into or enters natural water bodies after preliminary treatment in sewage treatment plants. One indirect source is tire wear particles (TWPs) produced by worn tires and ground weathering, where a small part (0.1–10.0%) is dispersed into the air. Most TWPs (90.0–99.9%) are released into water bodies through runoff or treated wastewater. Aging and weathering cause BTHs to precipitate and change chemical forms. Similarly, herbicides, insecticides, and antibacterial agents can enter the soil, water, and atmosphere [1]. BTHs are constantly accumulating in the environment due to their extensive use. Average BTH concentrations in atmospheric PM2.5 in Guangzhou and Shanghai, China, are 525 and 369 pg.m^−3^ [2]. In 51 cities, BTHs have been detected in both groundwater and surface water, with the average concentration reaching 406 ng·L^−1^ [3,4]. Twelve BTHs and eight related derivatives were detected in surface water, groundwater, rainwater, and suspended particles in the Guangzhou area. The groundwater concentration of BTHs was as high as 13.7 μg·L^−1^. The estimated maximum daily BTH intake of adults in Guangzhou was as high as 27.7 mg·d^−1^, which is higher than the limit set by the European Food Safety Authority [4,5]. In the United States, the median concentration of 5,6-dimethyl-benzotriazole detected in the air can reach 105 ng·m^−3^ [6]. In outdoor air samples from Spain, the highest concentrations of 1H-benzotriazole and 5-methyl-1H-benzotriazole were measured at 3.9 ng·m^−3^ and 2.9 ng·m^−3^, respectively [7]. Airport groundwater in North America exhibits extremely high concentrations of 1H-benzotriazole (126 mg·L^−1^) and 5-methyl-1H-benzotriazole (17 mg·L^−1^) [8]. Furthermore, an analysis of groundwater samples from across Europe revealed that out of 164 samples collected from various countries, the maximum concentrations observed were of 1H-benzotriazole (1032 ng·L^−1^) and a mixture of two isomers, 4-methyl-1h-benzotriazole and its isomer, 5-methyl-1h-benzotriazole (516 ng·L^−1^), with detection rates of approximately 53% and 52%, respectively [9]. In addition, BTHs are common contaminants in clothing; studies have shown that BTHs can be released from textile materials, penetrate the skin, and further enter the body [10].

With the widespread application and accelerated spread of BTHs in the environment, their potential toxic effects have attracted increasing attention. Studies have revealed a range of adverse effects associated with BTHs; for example, chronic exposure to personal care products (PPCPs) containing BTHs can affect neuroendocrine and neuronal development in juvenile Atlantic cod [11]. BTHs in tire particle leachate can cause growth inhibition in plants, abnormal fish embryo development, and death of water fleas [12]. Wastewater containing BTHs inhibits neurite growth in SH-SY5Y cells [13], causing neurotoxicity [14], and benzothiazole ionic liquids induce acute toxicity and tissue damage in zebrafish [15]. The inhibitory effect of BTHs on thyroid peroxidase derived from porcine thyroid is MBT = CMBT (5-chloro-2-mercaptobenzothiazole) > 2-ABTH > BTH [16]. Studies have shown that TWP leachate and wastewater containing a variety of BTHs have certain neurotoxic effects. The current comparative data on BTH toxicity are relatively limited, and the neurotoxicity of each BTH must be compared.

A comparative analysis of various BTHs was conducted between three relatively stable BTH derivatives (MBT, BTON, and 2-ABTH). All three structurally similar substitutions were carried out at the two-position of the thiazole ring in BTH (thiol group, hydroxyl group, and amino). BTH (74.1 ng·L^−1^) and 2-ABTH (0.21 ng·L^−1^) were detected in Guangzhou, BTON (4.9 ng·L^−1^) [3,4] groundwater. MBT in Brazil’s Tijuca Lagoon was as high as 2505 ng·L^−1^ [17]. BTH is mainly used as an intermediate for dyes, medicines, and rubber, with various pharmacological effects such as antibacterial, anti-inflammatory, anti-cancer, and anti-diabetic effects [18]. MBT is a widely used rubber accelerator and anti-aging agent, as well as a metal corrosion inhibitor and antibacterial agent [19]. BTON is used to prepare pyridinothiazole drugs, which have antibacterial, antifungal, and herbicidal properties [20]. 2ABTH is an impurity in drugs such as Pamiparol and can be used as a neutral carrier (ionophore) [21] to prepare polyvinyl chloride-based membranes.

Current toxicity studies have found that BTH has a 96 h LC_50_ for zebrafish (14.249 mg·L^−1^ = 105.4 μM) and a 96 h LC_50_ for sheephead minnow juveniles (41.9 mg·L ^−1^ ≈ 310.1 μM) [22]. MBT can induce oxidative stress in rainbow trout [23] and inhibit swim bladder inflation in zebrafish as a thyroid peroxidase (TPO) inhibitor [24]. It can also be used as a melanin inhibitor [25]. BTON has a 96 h LC_50_ of 358 μM in zebrafish. Oxidative stress negatively impacts embryonic development and reproduction [2]. In particular, 2-ABTH shows toxic effects [26]. As emerging environmental pollutants, research and reports on the developmental toxicity and neurotoxicity of the four BTHs are limited, and further research is needed. All four appear directly or indirectly in environmental water bodies through various migrations, and the neurotoxic effects of biological exposure have not been studied or reported in detail.

Zebrafish develop rapidly and are easy to observe, and their neural development is similar to that of mammals. Additionally, they are small in size, low cost, and used as an animal model to evaluate the neurodevelopmental toxicity of environmental pollutants [27,28]. The susceptibility of the nervous system to toxic damage varies by stage of development, and the embryonic stage is generally more sensitive than the adult stage, with zebrafish producing large numbers of transparent embryos that are easy to observe in vitro [29], while zebrafish embryos develop rapidly within 3 days of fertilization [30]. The book “*Neuromethods*” provides a comprehensive guide to Cell Culture Techniques, highlighting the utilization of zebrafish as a valuable tool for evaluating developmental neurotoxicity [31]. Specific methods include gene expression changes in developing zebrafish as potential markers for rapid developmental neurotoxicity screening [32]. Neurodevelopment of juvenile zebrafish can be evaluated using transgenic line zebrafish with fluorescent labeled tissue [27,33], and tests such as “fetal movement”, “light and dark tests”, and “startle response” are performed to assess possible behavioral changes resulting from neurotoxic exposure [34,35]. This study investigates the neurotoxic effects of different BTHs at the same concentration and explores possible toxicity mechanisms related to oxidation level indicators and measurements of gene expression.

## 2. Materials and Methods

### 2.1. Chemicals and Reagents

Benzothiazole (BTH, CAS: 95-16-9, purity 98%), 2-mercaptobenzothiazole (MBT, CAS: 149-30-4, purity 98%), 2-hydroxybenzothiazole (BTON, CAS: 934-34-9, purity 98%), and 2-aminobenzothiazole (2-ABTH, CAS: 136-95-, purity 97.0%) were purchased from McLin Reagent Company (Shanghai, China). The analysis included catalase (CAT), total superoxide dismutase (SOD), lipid peroxidation (MDA), and lipid peroxidase (SOD). The following reagents were purchased for the experiment: McLin Reagent Company (Shanghai, China); BAYOTEK (Shanghai, China) for catalase (CAT), total superoxide dismutase (SOD), lipid peroxidation (MDA), reactive oxygen species (ROS), and Enhanced BCA Protein Assay Kit; Invitrogen (Carlsbad, CA, USA) for TRIzol reagent, and Sigma (St. Louis, MO, USA) for MS-222 (ethyl 3-aminobenzoate, methane sulfonate) and dimethyl sulfoxide (DMSO).

### 2.2. Zebrafish Breeding and Embryo Collection

The Institute of Hydrobiology at the Chinese Academy of Sciences (Wuhan, China) provided adult wild-type (AB strain) zebrafish and transgenic zebrafish (HuC-GFP). The zebrafish were kept in a breeding system (Esen, Beijing, China) with a pH of 7.5 ± 0.5, a temperature of 27 ± 0.5 °C, and 14 h of light (08:00–22:00). They were fed freshly hatched brine shrimp twice a day (08:00 and 16:00).

The night before the experiment, male and female zebrafish were placed in breeding boxes in a 2:1 ratio to collect zebrafish embryos. For subsequent BTH exposure tests, fertilized eggs were collected the following morning within 4 h of fertilization and selected under a microscope. All animal experiments were conducted according to the Nanjing Institute of Environmental Sciences’ Guidelines for Care and Use of Laboratory Animals (IACUC-20200126). The test water used was tap water that had been aerated for more than 24 h.

### 2.3. Embryos Exposed to Different BTH Solutions

The embryos were randomly assigned to six-holes plates, with 10 embryos per hole. Each hole contained 10 mL of a 10 μM exposure solution of BTH, MBT, BTON, and 2-ABTH. The plates were incubated for 144 h at an exposure concentration of 10% BTH at 96 h LC50 (14.249 mg·L^−1^ = 105.4 μM). A BTH solution was chosen because previous toxicity studies showed that it does not produce an acute lethal effect on any of the four BTHs [2,22,23,24,25,26]. To maintain the solution concentration stability, the BTH solution was renewed daily. DMSO was used to dissolve the drug, resulting in a final exposure solution containing less than one ten-thousandth of DMSO. A DMSO solution of 0.1 mL·L^−1^ was used as a solvent control. A DMSO solution at this concentration has been proven safe and harmless to zebrafish [36]. Embryos were exposed at 6 days post-fertilization (dpf) and monitored twice daily throughout the experiment. Any deceased embryos or larvae were removed.

The developmental parameters of the surviving hatchlings, including the number of hatchlings, deaths, and body length N = 9), were recorded using a stereomicroscope (Nikon, SMZ25, Japan). The movements of 24 h post-fertilization zebrafish hatchlings were measured using an EthoVision XT 16 Animal Movement Tracking System (Noldus, Wageningen, The Netherlands) after being fixed with methylcellulose. The experiment was conducted in triplicate with 24 h post-fertilization zebrafish models (N = 15). The results were repeated independently at least twice.

### 2.4. Behavioral Experiments on Light and Dark Stimulation, Black and White Choice, and Tapping Stimulation of Zebrafish Larvae

Fertilized zebrafish embryos were exposed to 10 μM solutions of the four BTHs continuously for six days, as described in Section 2.3, to maintain a stable exposure. The observation area, detection procedure, and number of replicates were set according to Ref. [37] with 2 mL of water per hole. Data acquisition was performed using the animal movement trajectory tracking system EthoVision XT 16. The acquisition hardware of the animal trajectory tracking system EthoVision XT 16 consists of an infrared high-speed camera and board, which can accurately collect position information from zebrafish in dark environments. The software(EthoVision XT 16, version number: 16.0.1538) can also simultaneously analyze and calculate behavioral data.

#### 2.4.1. Light and Dark Stimulation Behavioral Test

At the beginning of the experiment, the zebrafish in the 24-hole plates were adapted to the bright environment. Setting the light program involved photoperiodic light and dark field stimulation (change between light and dark every 10 min, light/dark/light/dark) [38]. Each group of young zebrafish was tracked for 40 min using an EthoVision XT 16 to determine how far they moved, how long they stayed, and their swimming behavior. Under different lighting conditions, the total distance traveled, dwell time, and average speed of each group were calculated and analyzed statistically.

#### 2.4.2. Vibration Startle Response Test (VSRA)

For the VSRA, a DanioVision DVTD-0010 was used to deliver vibratory stimuli in a DanioVision observation chamber. The EthoVision XT 16 analyzed video tracking and escape responses. The DanioVision temperature control unit maintained a temperature of 28 °C. Tapping stimuli were used at intensity level 1, followed by a sequence of vibration stimuli at a fixed interstimulus interval (ISI). In each hole, one larva was placed in 2 mL of clear water. Before the first stimulation, juvenile fish were acclimated to the hole plate for 30 min. The videos were recorded at a rate of 25 frames per second. For VSR analysis, we measured motion speed (mm·s^−1^) within 2 s of pacing. We collected the motion trajectories of zebrafish larval groups within 80 s by tapping once every 30 s, cycling twice, and calculating the average speed of each fish group every 2 s for statistical analysis [39].

#### 2.4.3. Phototaxis Selection Behavior Test

**Figure 1:**
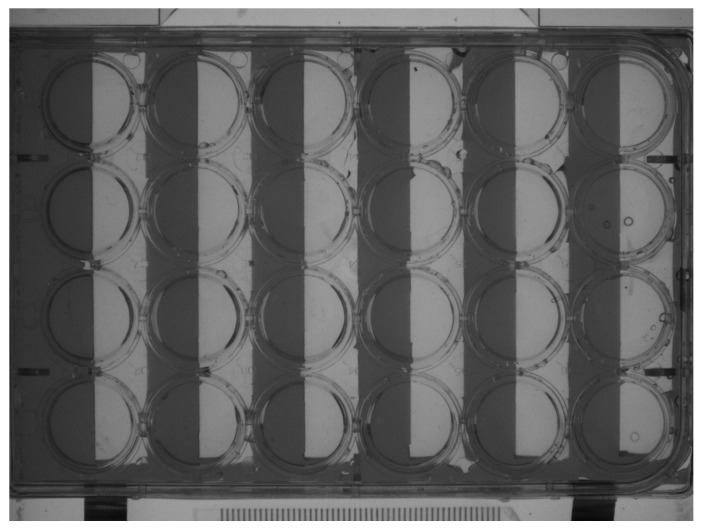
A black acrylic light shield is positioned beneath the 24-hole plate, which is cut to block half of the area of each observation hole. This configuration ensures that the white light from the bottom layer cannot irradiate the zebrafish through the light shield. The infrared plate at the bottom of the observation box is capable of emitting infrared rays, which can penetrate the acrylic plate but not the zebrafish. Consequently, the infrared high-speed camera is able to capture the movement of the zebrafish in the black area without light and in the area with light.

Based on the phototaxis of zebrafish, a light-shielding plate was used to block half of each hole in the 24-hole plate so that each hole was divided into black and white parts, as shown in Figure 1 [40]. Software was used to deduce the movement, distance, travel time, and swimming behavior trajectory of each group of zebrafish larvae within 40 min. After calculating the total passage distance, residence time and average speed of each group of fish under different lighting conditions, the data were statistically analyzed.

### 2.5. Image Observation of Transgenic Zebrafish

According to previous methods, zebrafish embryos carrying the HuC-GFP transgenic line were exposed to 10 µM solutions of four BTHs [38]. Section 2.3 describes stable BTH exposure. The transgenic line of HuC-GFP zebrafish has central nervous system GFP expression integrated into the promoter sequence of the elavl3 gene. Elavl3 encodes HuC, a neuron-specific RNA-binding protein and one of the earliest neuronal markers in zebrafish. We observed central nervous system expression and the motor nerve at 72 hpf following the method described in a previous study [38]. After treatment with four BTHs (10 μM) at 72 hpf, 10 zebrafish larvae from each group were selected and fixed with 4% paraformaldehyde for 0.5 h. Images of the central nervous system were obtained using stereofluorescence microscopy (Nikon, SMZ25, Tokyo, Japan) in the HuC-GFP transgenic line.

### 2.6. Quantitative qPCR Detection

Total RNA was extracted from groups of 50 zebrafish juveniles in different treatment groups (n = 3) using TRIzol reagent. The RNA concentration was determined and reverse transcribed into cDNA using PrimeScript RT. The housekeeping gene β-actin served as an internal control. Analysis was performed using the 2^−ΔΔCt^ method. After evaluating the neurotoxicity and developmental toxicity of BTHs in juvenile zebrafish, we identified a set of neurodevelopment-related genes and oxidative stress-related genes for further investigation. The primer sequences for the selected genes are provided in Table 1. Each sample was tested in triplicate for accuracy.

### 2.7. Assessment of Oxidative Stress Levels

Fertilized zebrafish embryos were exposed to 10 μM solutions of four BTHs for 6 days, maintaining stable BTH exposure, as described in Section 2.3. Homogenates were analyzed for SOD, MDA, GSH, and CAT in zebrafish larvae randomly selected from each treatment group (50 larvae pooled into one sample, N = 3). After calibrating the concentration using an enhanced BCA protein detection kit, we determined the activity levels of SOD, MDA, GSH, and CAT according to the kit instructions and performed statistical analyses of the data in GraphPad Prism 9.5.

## 3. Results

### 3.1. Developmental Toxicity of BTHs to Zebrafish Juveniles

**Figure 2:**
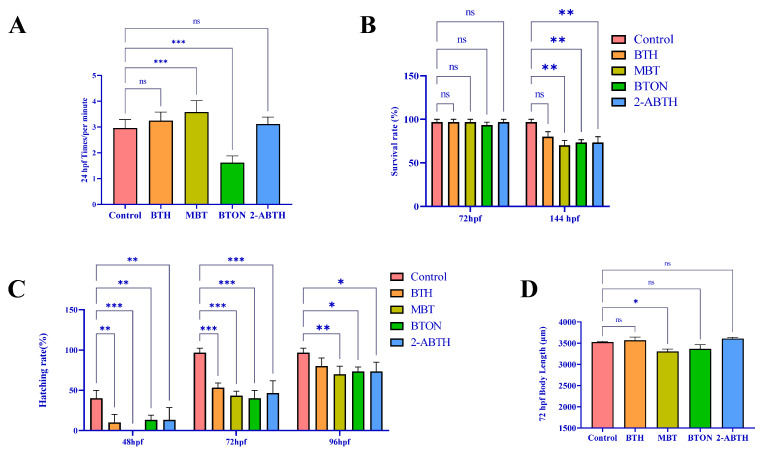
BTH effects on developmental toxicity of zebrafish. All data are expressed as mean ± SE. (**A**) Spontaneous movement, N = 45; the number of times the zebrafish curls and wriggles inside the embryo. (**B**) Survival rate at 72 and 144 hpf after fertilization, N = 3. (**C**) Hatching rate at 48–96 hpf after fertilization, N = 3. (**D**) Body length at 72 hpf after fertilization, N = 9. Some of the unhatched embryos were measured after the egg membranes were peeled off using dissecting forceps under a microscope, and the larvae were allowed to stretch for 2 h. Furthermore, because some zebrafish embryos exposed to BTHs did not survive to 144 hpf, body length was not compared for this time period. ^ns^ *p* < 0.12, * *p* < 0.033, ** *p* < 0.002, *** *p* < 0.001, compared to control.

After exposure to a 10 μM solution for 24 h, zebrafish embryos exhibited spontaneous changes in movement. Compared to the control group, the MBT-exposed group showed enhanced movement, whereas the BTON-exposed group showed inhibited movement, as shown in Figure 2A. Unlike the control group, zebrafish larvae exposed to BTH did not die at 72 hpf, as shown in Figure 2B. Moreover, Figure 2C shows that zebrafish larvae in the BTH-exposed group exhibited delayed hatching at 48–72 hpf compared to the control group. It is worth noting that all surviving embryos hatched after 96 hpf, with the MBT group having the greatest delay in hatching. Moreover, it appears that there was no significant change in zebrafish larvae’s body length at 72 hpf. It is worth noting that the overall trend observed was similar to that of the hatching level, as illustrated in Figure 2D.

### 3.2. Behavioral Changes in Zebrafish Juveniles

#### 3.2.1. Light and Dark Stimulation

**Figure 3:**
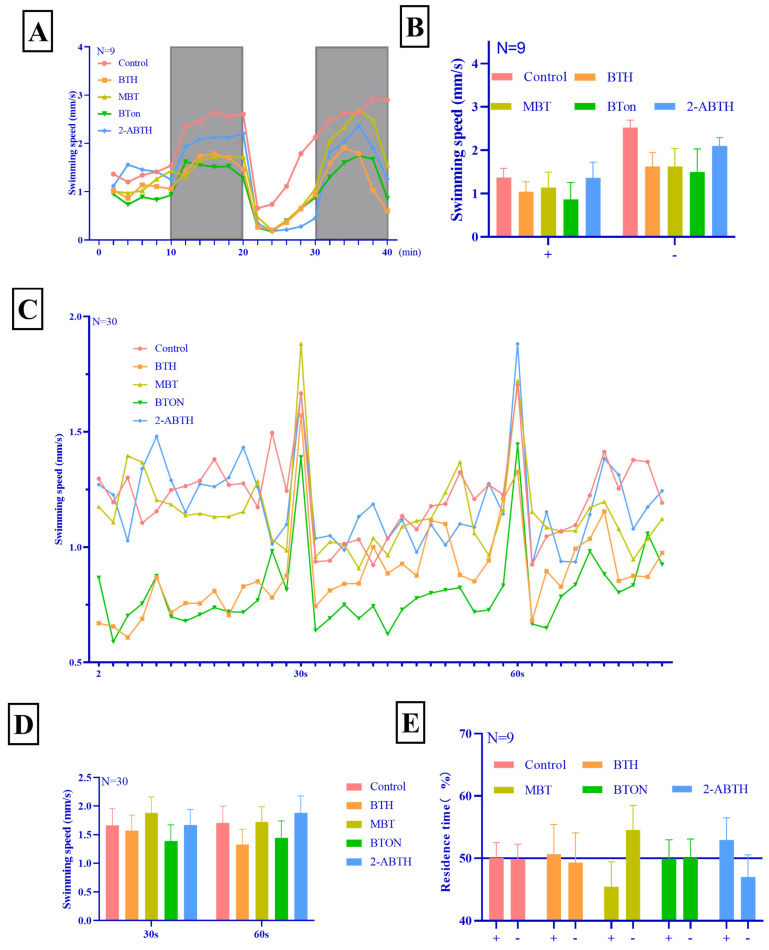
BTH effects on zebrafish behavior. The data are presented as mean ± SE. (**A**) Mean velocity change of zebrafish under light and dark stimuli, N = 9. The white background area indicates that the zebrafish received light during the time period in question, while the gray background represents those that did not receive light and were in a dark environment during the same period. (**B**) The mean velocity of the zebrafish was observed under light and dark environments, N = 9. The symbol “+” indicates that the zebrafish received light in a bright environment during the observation period, while the symbol “−” denotes the absence of light in a dark environment. (**C**) The vibration startle response test involved velocity fluctuation, N = 30. Two knocks were performed at seconds 30 and 60, with the two highest peaks on the graph representing the instantaneous mean velocity of the zebrafish during the two seconds in which it was stimulated to undergo rapid swimming. (**D**) Comparison of instantaneous mean velocities of zebrafish during two seconds of stimulated fast swimming at 30 and 60 s, N = 30. (**E**) residence time of zebrafish in light and dark areas, N = 9. The symbol “+” indicates that the zebrafish stayed in the semicircular observation area that was illuminated by light without a visor, and the symbol “−” indicates that it stayed in the dark semicircular observation area that was obscured by a visor.

Figure 3A,B demonstrate that zebrafish larvae swim faster in dark environments than in bright ones. In 0–10 min, the speed of both the treatment group and the control group was about the same because they both adapted to the bright environment before data collection. In bright conditions, the BTHs and control groups did not differ significantly. However, in dark environments, the BTH group exhibited lower swimming speeds than the control group to varying degrees.

#### 3.2.2. Vibration Startle Response Test

In a dark environment, the swimming speeds of BTH and BTON were relatively low compared to that of the control group, and MBT and 2-ABTH were not significantly changed, as shown in Figure 2C,D. However, the speeds of all four BTHs at these two knocks surged to similar speeds with no statistical difference. The four BTHs did not significantly affect the zebrafish larvae’s physical locomotor ability, and the decrease in locomotor speed may be related to neural development.

#### 3.2.3. Phototaxis Test

Figure 3E shows that the control, BTH, and BTON groups did not exhibit a significant preference for light or dark environments. By contrast, MBT preferred dark environments and 2-ABTH preferred bright environments. According to the literature, MBT inhibits melanin production, resulting in decreased eye pigmentation and increased sensitivity to light, which may explain the preference for darkness observed in the experiment [25]. As described in Section 3.2.1, 2-ABTH was more active in bright environments than the other groups, as confirmed by the phototaxis test.

### 3.3. Influence on Central Nervous System Development

**Figure 4:**
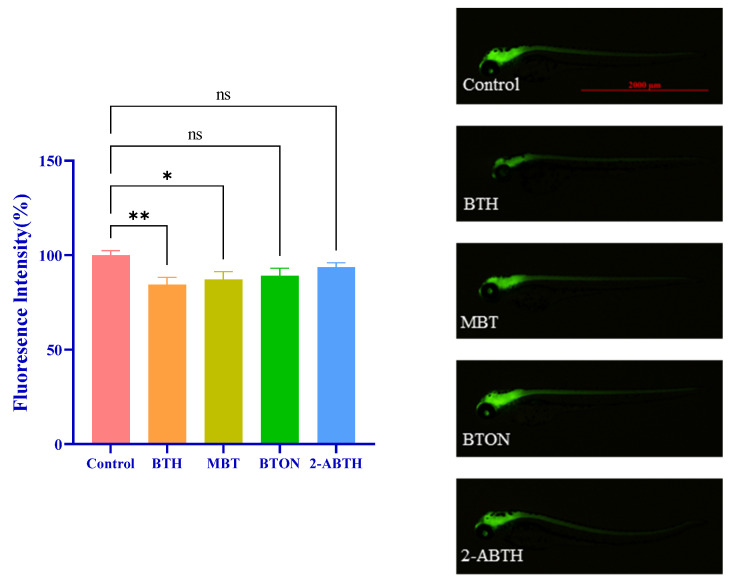
Normalized comparisons were made with the average fluorescence intensity of the control group.Analysis of BTH’s impact on the fluorescence of the central nervous system of Tg (HUC-GFP) zebrafish. The image on the right shows the green fluorescence of Tg (HUC-GFP) zebrafish under fluoroscopy in response to blue laser excitation, reflecting the development of the central nervous system, with stronger fluorescence representing more nerve cells. The results are presented as mean ± SE. (n = 9). ^ns^ *p* < 0.12, * *p* < 0.033, ** *p* < 0.002, compared to control.

Figure 4 shows a notable variation in the fluorescence intensity of zebrafish larvae’s central nervous system, specifically in the BTH and MBT exposure groups (*p* < 0.033, *p* < 0.002), 72 h after exposure to the BTH solution.

### 3.4. Effects on the Antioxidant Systems of Zebrafish

**Figure 5:**
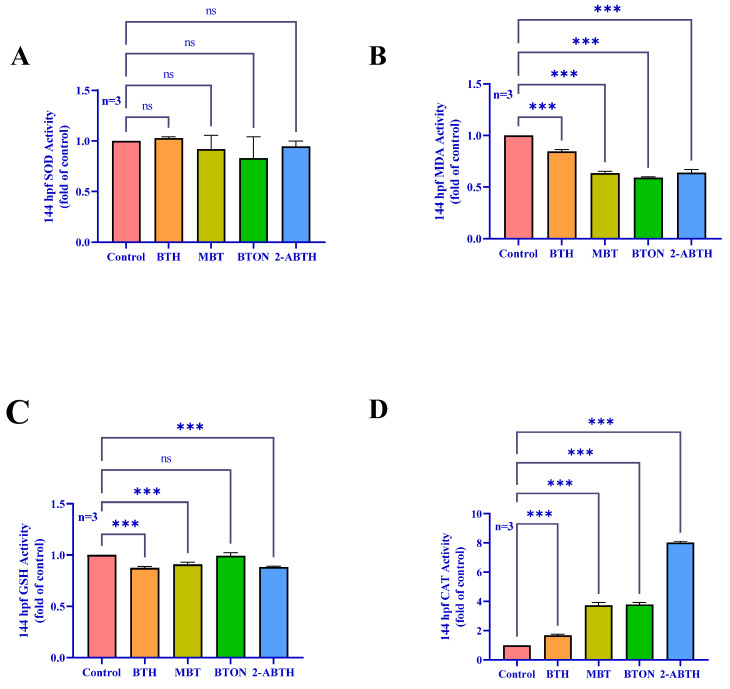
The effects of BTH on the enzyme-related activities of the zebrafish antioxidant system were normalized and compared with the enzyme activities of the control group. (**A**) CAT (catalase) enzyme activity, (**B**) SOD (superoxide dismutase) enzyme activity, (**C**) MDA (glutathione) enzyme activity, (**D**) GSH (glutathione) enzyme activity. ^ns^ *p* < 0.12, *** *p* < 0.001, compared to control.

The SOD content of zebrafish larvae exposed to the BTH solution did not show a significant effect compared to the control group (Figure 5A) (*p* < 0.12). However, MDA levels were significantly lower than in the control group (Figure 5B) (*p* < 0.001). Our study findings indicate that CAT content was significantly higher in the experimental group than in the control group (Figure 5D) (*p* < 0.001). Moreover, the GSH content of the three drugs, BTH, MBT, and 2-ABTH, was significantly lower than in the control group (Figure 5C) (*p* < 0.001). These findings suggest that BTHs can detrimentally affect zebrafish larvae’s antioxidant systems.

### 3.5. Gene Expression Related to Neurodevelopment in Zebrafish Larvae

**Figure 6:**
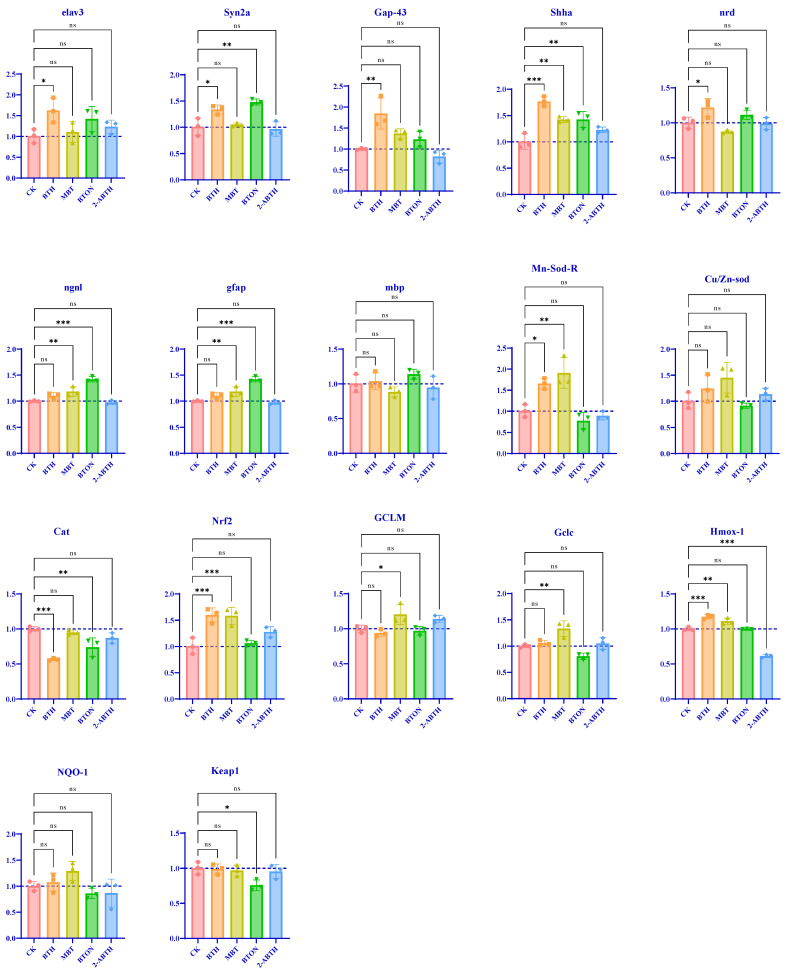

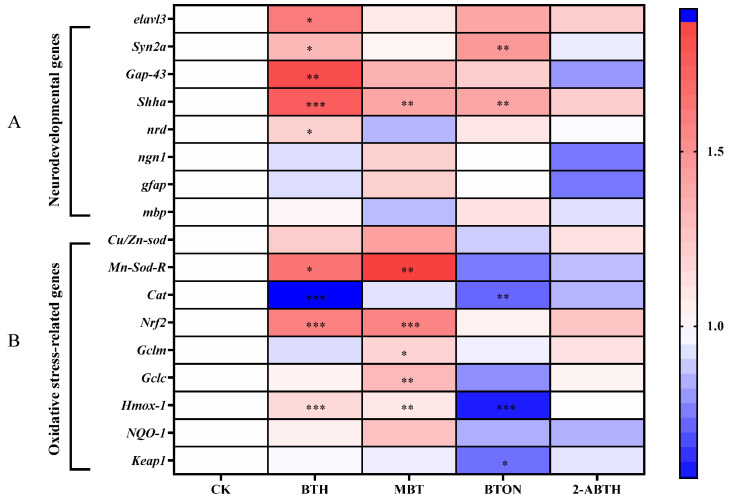
Comparison plot of the effects of BTHs on zebrafish-related gene expression. All gene expression was normalized to the control group for comparison, with a y = 1 dotted line on the y-axis. Below the dotted line indicates that the expression of this gene was suppressed compared to the control group, and above the dotted line indicates that this gene was overexpressed compared to the control group. The following heatmaps are shown in green for suppressed gene expression and in red for overexpression: (**A**) heatmap of neurodevelopment-related gene expression; (**B**) heatmap of oxidative stress-related gene expression. ^ns^ *p* < 0.12, * *p* < 0.033, ** *p* < 0.002, *** *p* < 0.001, compared to control.

Figure 6A illustrates alterations in gene expression that occurred during neurodevelopment after exposure to BTHs. *Elavl3*, *Syn2a*, and *nrd* were slightly upregulated (*p* < 0.01), *Gap-43* was upregulated (*p* < 0.002), and Shha was significantly upregulated (*p* < 0.001) in the BTH group compared to controls. In the MBT group, Shha was upregulated compared to the control group (*p* < 0.002). Similarly, both *Syn2a* and *Shha* were upregulated in the BTON group compared to the control group (*p* < 0.002). No statistically significant differences were found for the remaining genes.

Neurodevelopment is shown in Figure 6B. In the BTH group, Mn-Sod showed a slight increase in expression (*p* < 0.01), while *Nrf2* and *Hmox-1* were significantly upregulated (*p* < 0.001). CAT was significantly downregulated (*p* < 0.001) compared to the control group. In the MBT group, Gclm showed slightly increased expression (*p* < 0.01), and *Mn-Sod*, *Gclm,* and *Gclc* were upregulated (*p* < 0.002) compared to the control. In the BTON group, *Keap1* was slightly downregulated (*p* < 0.01) compared to the control group. CAT was downregulated (*p* < 0.002) and *Hmox-1* was significantly downregulated (*p* < 0.001). No statistical differences were observed for the remaining factors.

According to these findings, BTHs appear to negatively affect zebrafish’s neurodevelopment and antioxidant systems.

## 4. Discussion

With the large-scale use of BTHs, harmful components of new pollutants [1], concerns about the safety of other BTHs have been raised. A variety of BTHs have been detected in the atmosphere, soil, and water of many cities in China [3,4]. However, there are few corresponding toxicity studies and management regulations lack reference data. Hence, there is an urgent need to study their toxicological properties.

This study investigated the developmental and neurotoxic effects of four BTHs using zebrafish embryos as an animal model. At 24 h post-fertilization, zebrafish embryos exhibited spontaneous locomotion [41], potentially affected by chemical exposure and requiring further investigation [42]. MBT significantly affected the spontaneous movement of zebrafish embryos, whereas BTON had a significant inhibitory effect. At the same time, zebrafish larvae exposed to BTHs showed delayed hatching at 48–72 hpf, and MBT significantly inhibited hatching at 48 hpf. At a concentration of 10 μM, none of the four BTHs significantly affected zebrafish embryo survival at 72 hpf. Our results suggest that BTHs have a notable effect on zebrafish embryonic development at non-harmful levels and do not have immediate toxic effects. Swimming behavior has been extensively used to assess the neurotoxicity of environmental chemicals because it is a key indicator of neurodevelopment in the early stages of zebrafish development [43]. In this study, the four BTHs showed differences in behavior compared to the control group. However, these differences were not statistically significant, which is consistent with related research [26]. The vibration startle response test in this study indicated that the four BTHs did not significantly affect zebrafish larvae’s bodily movement. However, differences in movement speed may be related to neural development.

It was hypothesized that BTHs may be linked to neurodevelopmental disorders in zebrafish larvae. To test this hypothesis, the effects of four BTHs on neurodevelopment were evaluated using the transgenic line HuC-GFP in transgenic zebrafish. The study found that exposure to BTH resulted in reduced green fluorescence intensity in the brain and spinal cord of HuC-GFP transgenic zebrafish (*p* < 0.033). Additionally, MBT inhibited the fluorescence intensity of zebrafish (*p* < 0.002). The fluorescence intensity of 2-ABTH did not change significantly, and the experimental results were consistent with the literature [26]. The results showed that BTH and MBT may affect central nervous system development, potentially leading to neurotoxicity. BTON and 2-ABTH did not significantly affect the central nervous system development of zebrafish at a concentration of 10 μM.

The expression levels of neurodevelopment-related genes were measured using real-time quantitative fluorescence PCR to better understand the effects of the four BTHs on zebrafish neuronal damage. *Elav3* is an early neuronal marker in zebrafish that plays a critical role in controlling neuronal differentiation and maintenance [44,45], BTH caused a slight upregulation of *elav3* expression (*p* = 0.05), possibly due to neuronal damage promoting neuronal differentiation. *Syn2a* regulates neuron-specific synaptic vesicles and neurotransmitter release during central nervous system development. *Syn2a* upregulation may impact synaptic growth and neuronal differentiation. The results suggest slight upregulation of BTH (*p* = 0.01) and BTON (*p* = 0.001), potentially indicating synaptic growth inhibition and neuronal differentiation. *Gap43* is a specific cytoplasmic protein found in nerve tissue. It is used as a marker for axonal growth reinduction during nerve regeneration after injury [46,47]. In zebrafish larvae exposed to chemicals such as BDE-47 [48], which suppress axonal growth, gap43 gene expression may increase as a compensatory mechanism, as demonstrated by the decrease in fluorescence intensity of HuC-GFP transgenic zebrafish exposed to BTH. *Shha* plays a crucial role in different organ systems, particularly the nervous system. It is vital for neural tube patterning, neural stem cell proliferation, and neuronal and glial cell survival. Overexpressing Shha through BTH, MBT, and BTON increases neuronal cell survival, protects against neurotoxicants, and suppresses neuronal precursors such as neuron-restricted precursors (*p* < 0.001, *p* = 0.003, and *p* = 0.003, respectively) [49,50]. Ngn1 encourages neuronal cell differentiation into neurons and low transcript levels hinder neuron generation [51]. Nrd also enhances neuronal differentiation after Ngn1 expression and supports photoreceptor cell proliferation in the zebrafish retina [52,53]. *Gfap* is a member of the intermediate filament structural protein family and is highly expressed in differentiated glial cells [50]. The transcription level of *mbp* influences the production of myelin basic protein, which is a crucial component of myelin in both the central and peripheral nervous systems [54,55]. When transcript levels are low, nervous system production may decrease. Our results confirm that exposure to BTHs inhibits neuronal development in the zebrafish central nervous system.

Nerve damage often occurs simultaneously with oxidative damage. This study detected four oxidative damage markers (SOD, MDA, GSH, and CAT), except GSH enzyme activity in the BTON group, which did not exhibit a statistically significant change (*p* < 0.12). Other BTH zebrafish showed a significant reduction in MDA and GSH enzyme activities (*p* < 0.001) as well as an increase in CAT enzyme activity (*p* < 0.001). SOD and CAT are important enzymes that work together to reduce ROS. The effects were consistent with experimental results of oxidative damage [15]. These results indicate that BTH-induced oxidative stress may impact zebrafish neurodevelopment. Observed changes in antioxidant enzyme activities and accumulation of free radicals in juvenile zebrafish may indicate potential oxidative damage.

The expression of genes related to oxidative levels was measured to further understand the oxidative damage mechanism in zebrafish caused by the four BTHs. In zebrafish exposed to BTH and MBT, *Mn-Sod* gene expression was upregulated compared to the control group (*p* < 0.01 and *p* < 0.001). Additionally, *Cu/Zn-Sod* was present in the cytoplasm and intermembrane space of mitochondria. *Mn-Sod* mainly exists in the mitochondrial matrix. Its main function is to protect mitochondrial components from harmful superoxide free radicals and upregulate genes indicating oxidative damage. Secondly, *the Nrf2* gene expressions of BTH and MBT were significantly upregulated (*p* < 0.001), and *Nrf2* regulates the expression of multiple genes [56]. *Nrf2* controls the key component glutamate cysteine ligase (GCL) in the endogenous antioxidant system. GCL catalyzes the rate-limiting step in glutathione synthesis and consists of catalyst (*Gclc*) and modifier (*Gclm*) subunits. *Gclc* expression is induced by *Nrf2*, emphasizing the regulatory pathway’s importance in glutathione synthesis [57]. *Nrf2* also controls glutathione peroxidase (GPX) 2 expression, producing oxidized glutathione (GSSG) and glutathione reductase (GSR) 1 while reducing peroxide and GSSG, thereby maintaining reduced glutathione (GSH) levels in cells [58]. Exposure to low concentrations of electrophilic chemicals depleted GSH, and overexpression of Nrf2 in zebrafish with BTH and MBT (*p* < 0.001) significantly decreased GSH content (*p* < 0.001). Nrf2 activation leads to Keap1 dissociation and facilitates Nrf2 nuclear translocation, which activates antioxidant response elements located in the enhancer region of Nrf2-regulated genes [59]. Keap1 expression is regulated by Nrf2, which induces Keap1 as a negative feedback loop [60]. *Hmox-1* and *NQO-1* are proteins that protect cells from oxidative stress damage. Upregulation of these proteins significantly reduces inflammation and improves survival during severe infection [59,61]. *Nrf2* expression was significantly reduced in the BTON group, which significantly inhibited *Hmox-1* (*p* < 0.001). However, GSH did not show any significant changes. CAT was significantly inhibited in both the BTH (*p* < 0.001) and BTON groups (*p* = 0.04). Downregulation of *CAT* expression levels led to an increase in CAT enzyme activity [62]. These observations are consistent with our measurement results. Our results confirm BTH’s ability to induce oxidative damage in zebrafish larvae at the genetic level, indicating that BTH exposure can cause oxidative damage. Although there were no significant changes in 2-ABTH-related genes at a concentration of 10 μM, long-term exposure (120 hpf) to 2-ABTH at a concentration of 0.3 μM produced significant oxidative damage zebrafish brains [26], possibly related to antioxidant stress and cell protection. Changes in key genes such as *Mn-sod*, *Nrf2,* and *Hmox-1* suggest that zebrafish initiate a defensive oxidative stress response when exposed to BTHs.

Our study findings suggest that BTHs can potentially cause nerve and oxidative damage through various mechanisms. The regulation of antioxidant genes may lead to oxidative stress as a result of BTHs. The study examined the effects of BTH exposure on central nervous system development in HuC-GFP transgenic zebrafish. Our results showed that their antioxidant defense system was destroyed and their oxidation levels increased. These findings highlight the critical role of oxidative stress and shed light on BTH neurotoxicity mechanisms in zebrafish.

## 5. Conclusions

The exposure to BTHs may indirectly impact the development of the central nervous system by disrupting antioxidant defenses and elevating oxidation levels. Zebrafish embryos exposed to 10 μM of BTHs for 24 h showed that MBT promoted spontaneous movements, whereas BTON inhibited them. Zebrafish larvae exposed to BTHs showed delayed hatching at 48–72 hpf, with MBT significantly inhibiting hatching at 48 hpf. The survival rates of all four BTHs exposed to 72 hpf were not significantly affected. Exposure to BTH and MBT resulted in reduced HuC-GFP green fluorescence intensity in the brain and spinal cord of transgenic zebrafish, indicating neurotoxicity and an impact on central neural development. Differences in zebrafish behavior were observed after exposure to BTHs at 144 hpf, attributed to neurodevelopmental deficits caused by exposure. The neurotoxicity of BTH was demonstrated by the upregulation and resulting neuronal damage of genes such as *elavl3*, *Gap-43, Shha*, and *nrd*. Additionally, MBT caused oxidative damage through the overexpression of *Gclc* and *Gclm* genes, which are two motifs of glutamate cysteine ligase. It also depleted and decreased GSH content. Furthermore, MDA inhibited CAT expression, leading to an increase in CAT enzyme activity. Meanwhile, the enzyme activity assay demonstrated that all four BTHs caused significant oxidative damage. Results comparing the toxicity of the four BTHs at a 10 μM concentration are as follows:

Developmental toxicity: MBT > BTON ≈ 2-ABTH > BTH
Neurotoxicity: BTH > MBT > BTON ≈ 2-ABTH
Oxidative damage: MBT > BTH > BTON > 2-ABTH

## Figures and Tables

**Table 1:** Sequences for real-time quantitative PCR primers.

Gene Name	Primer Sequences	
	Positive	Reverse
*β-actin*	ACAGGGAAAAGATGACACAGATCA	CAGCCTGGATGGCAACGTA
*elavl3*	AGACAAGATCACAGGCCAGAGCTT	TGGTCTGCAGTTTGAGACCGTTGA
*Syn2a*	GTGACCATGCCAGCATTTC	TGGTTCTCCACTTTCACCTT
*Gap-43*	TTAACGGAGGACCAGTGCAA	GACGGAGGTCTGAGTCCTGA
*Shha*	AGACCGAGACTCCACGACGC	TGCAGTCACTGGTGCGAACG
*nrd*	CAGCAAGTGCTTCCTTTTCC	TAAGGGGTCCGTCAAATGAG
*ngn1*	TGCACAACCTTAACGACGCATTGG	TGCCCAGATGTAGTTGTGAGCGAA
*gfap*	GGATGCAGCCAATCGTAAT	TTCCAGGTCACAGGTCAG
*mbp*	AATCAGCAGGTTCTTCGGAGGAGA	AAGAAATGCACGACAGGGTTGACG
*Cu/Zn-* *Sod*	GTCGTCTGGCTTGTGGAGTG	TGTCAGCGGGCTAGTGCTT
*Mn-Sod*	GTCCGCACTTCAACCCTCA	TCCTCATTGCCACCCTTCC
*Cat*	TGATCTTAGCAAATGCAACACTGA	TGCAAAGGCCCCCATTTT
*Nrf2*	GACAAAATCGGCGACAAAAT	TTAGGCCATGTCCACACGTA
*Gclm*	AAGCCAGACACTGACACACC	ATCTGGAGGCATCACACAGC
*Gclc*	CTCCTCACAGTCACGGCATT	TGAATGGAGACGGGGTGTTG
*Hmox-1*	ACGCTTACACCCCGCTACCTC	ATCCCCTTGTTTCCAGTCAG
*NQO-1*	CCTGCCATTCTGAAAGGCTGGT	GTGGTGATGGAAAGCACTGCCT
*Keap1*	CCAACGGCATAGAGGTAGTTAT	CCTGTATGTGGTAGGAGGGTT

## Data Availability

The data presented in this study are available upon request from the corresponding author.
